# T_1_ mapping for characterization of myocardial fibrosis in hypertrophic cardiomyopathy

**DOI:** 10.1186/1532-429X-18-S1-P320

**Published:** 2016-01-27

**Authors:** Ying Liu, Shun Qi, Zhankui Wang, Jianmin Zheng, Tianjing Zhang, Andreas Greiser, Hong Yin

**Affiliations:** 1 Dept. of Radiology, Xijing Hospital, Fourth Military Medical University, Xi'an, China; 2 Siemens Healthcare, MR Collaborations NE Asia, Beijing, China; 3 Siemens Healthcare, Erlangen, Germany

## Background

It was reported that the occurrence of myocardial fibrosis was related to sudden cardiac death and heart failure in patients with HCM. Late gadolinium enhancement (LGE) magnetic resonance imaging (MRI) is a widely used clinical method to detect fibrosis, however, its spatial resolution is limited. The purpose of this feasibility study is to determine the value of T_1_ mapping for the non-invasive assessment of myocardial fibrosis in patients with hypertrophic cardiomyopathy (HCM).

## Methods

Thirty HCM patients and 20 healthy volunteers underwent conventional late gadolinium enhancement (LGE) imaging and T_1_ mapping (Siemens prototype sequence) on a clinical 1.5T scanner (MAGNETOM Aera, Siemens Healthcare). T_1_ mapping was performed with a modified look-locker inversion-recovery (MOLLI) sequence acquired during breath hold in the same planes after LGE imaging. Typical imaging parameters were: non-selective inversion pulse, steady-state free precession single-shot read out in mid-diastole, FOV of 360 × 250 mm^2^, matrix of 192 × 150, slice thickness of 8 mm, TR/TE of 375.28/1.01 ms, minimum inversion time of 90 ms, inversion time increment of 80 ms, FA of 35^?^, PAT factor of 2, number of inversions 2, images acquired after first inversion 3, pause 3 heart beats, and images acquired after second inversion. T_1_ Mapping was used for measurement of T_1_ values. Global ECV values were calculated from T_1_ maps acquired pre- and post-contrast calibrated by blood hematocrit. The extracellular volume (ECV) value was calculated as: ECV = (1-hematocrit) (1/T1myopost-1/T1myopre) / (1/T1bloodpost-1/T1bloodpre).

## Results

**Figure 1:**
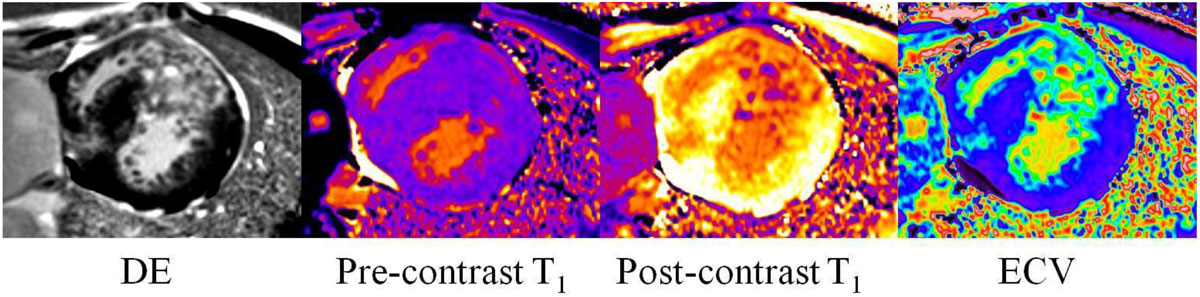
Figure 1

HCM patients had the same age as control group (38.2 ± 18.5 vs. 34.8 ± 15.7, P=NS). Pre-contrast myocardial T_1_ time and myocardial ECV in patients with HCM was significantly higher than the measurement in control cases, and post-contrast myocardial T_1_ time in HCM patients was significantly lower than that in control cases (*P* < 0.05, respectively) (Table 1, Figure 1).

**Table 1:** T1 measurement and ECV in cases with HCM and in control cases

Mapping parameters	HCM	Control	P value
Pre-contrast T1 value	1217.3 ± 97.4	1143.6 ± 84.3	0.021
Post-contrast T1 value	489.3 ± 57.1	503.1 ± 64.4	0.016
ECV	0.257 ± 0.036	0.231 ± 0.028	0.009

## Conclusions

The MOLLI T_1_ mapping used in the present study has combined the fast acquisition time and multiple contrast capabilities of the TI scout with the improved CNR, spatial resolution, and spatial coverage of the segmented IR LGE scans. The combination reduces the total imaging time, enables T_1_ quantification, and does not compromise image quality. A relatively higher pre-contrast T_1_ value and ECV, and lower post-contrast T_1_ value were found with T_1_ mapping in the myocardium of HCM patients, which suggested T_1_ mapping is better in the evaluation of myocardial fibrosis. The main limitation is that we had a small patient population without subgroup analysis and follow-up.

